# Workplace violence and its associated factors among nurses working in public hospitals of eastern Ethiopia: a cross-sectional study

**DOI:** 10.1186/s12912-022-01078-8

**Published:** 2022-11-07

**Authors:** Henok Legesse, Nega Assefa, Dejene Tesfaye, Simon Birhanu, Seid Tesi, Fenta Wondimneh, Agumasie Semahegn

**Affiliations:** 1 School of Nursing and Midwifery, College of Health and Medical Sciences, Haramaya University, Harar, Ethiopia; 2 Department of Population, Family and Reproductive Health, School of Public Health, University of Ghana, Accra, Ghana

**Keywords:** Workplace violence, Nurses, Public hospitals, Eastern Ethiopia

## Abstract

**Background:**

Workplace violence is one of the global health concerns. Although nurses are the backbone of the health care provision, they are highly subjected to workplace violence in healthcare. Nevertheless, there is a paucity of evidence on the extent of workplace violence against nurses in Ethiopia in general and Eastern Ethiopia in particular. Hence, this study aimed to assess the extent of workplace violence against nurses and its associated factors among nurse professionals working at public hospitals in eastern Ethiopia.

**Methods:**

Hospital-based cross-sectional study was conducted among 603 nurses working in public hospitals in eastern Ethiopia. Nurses were recruited using a simple random sampling method at their workplace (health facilities). A pretested self-administered questionnaire was used to collect data. Descriptive, binary and multivariable logistic regression analyses were performed. The adjusted odds ratio (AOR) with a 95% confidence interval (CI) was used to declare significant association.

**Results:**

Among the 620 estimated sample**,** 603(97.3%) of the nurses gave consent and completed the self-administered questionnaire. The prevalence of workplace violence against nurse professionals in the last 12 months was 64.0% (95%CI: 60.2–67.7%). Nurses who were working in surgical (AOR: 2.30, 95%CI: 1.01–5.26), psychiatric (AOR: 3.06, 95%CI: 1.11–8.46), emergency (AOR: 3.62, 95%CI: 1.46–8.98), and medical wards (AOR: 5.20, 95%CI: 2.40–11.27); being worried of workplace violence (AOR: 1.71, 95%CI: 1.09–2.69); witnessed of physical workplace violence (AOR: 5.31, 95%CI: 3.28–8.59); claimed “absence/not-aware” of reporting procedure on workplace violence (AOR: 2.24, 95%CI: 1.45–3.46); and claimed “absence/not-aware” of institutional policies against workplace violence (AOR: 2.68, 95%CI: 1.73–4.13) were factors associated with nurses’ experience of workplace violence in eastern Ethiopia.

**Conclusions:**

Workplace violence against nurses was found to be unacceptably high in the study area (eastern Ethiopia). We suggest that stakeholders could work on early risk identification and management of violent incidents, establish violence reporting and sanction mechanisms using contextual strategies to prevent workplace violence against nurse professionals.

**Supplementary Information:**

The online version contains supplementary material available at 10.1186/s12912-022-01078-8.

## Background

Workplace violence (WPV) is defined as “The intentional use of power, threatened or actual, against another person or a group, in work-related circumstances” [1]. The WPV inflicted injury could be physical, psychological or concurrent in form. Physical and psychological WPV results in several consequences, including disturbing emotion, burnout, job dissatisfaction, substance addiction and other psychological effects, which ultimately endangers the victims’ wellbeing and results in poor performance and lost productivity [2, 3]. WPV greatly impacts the communication line between patients and health workers in that the victims behave “patient-avoiding behaviors” and distance from talking with or listening to the patients’ need and concerns [2, 4]. Especially in sub-Saharan Africa, due to compromised health policy and practice and the emigration of health professionals, it is challenging to retain healthcare task forces. This challenge is to an extent linked with WPV [4].

Nurses are working as first-line care providers and are the largest task forces working in different varieties of working settings, they are highly victimized by WPV [5, 6]. It continues as a growing global concern in health care institutions [7–9]. Nearly three-fourths (72%) of nurses reported that they do not feel safe in the workplace [10]. The extent of WPV varies from place to place ranging from 24.7% to 88.9%. It is serious in an emergency, geriatric and psychiatry departments of the health facilities [9, 11]. Even though, different studies indicate that violence against Nurses is growing, 80% of workplace assaults among registered nurses went unreported formally [12]. Lack of reporting mechanism, and policy framework; lack of trust in the management system or fear of being blamed were some among the reasons for underreporting of the incidents [13]. Colleagues, supervisors/directors, physicians, patients and patients’ relatives were the primary perpetrators of WPV [9, 11].

A piece of literature on workplace violence among nurses in Africa includes, a cross-sectional study among nurses in four hospitals and eleven primary health care centers was conducted in Egypt. The result was 27.7% reported violence of any form and specifically 69.5% verbal & 9.3% physical violence was noted [14] Another study conducted in Malawi, WPV among the nurses was 71%. The type of violence experienced was physical (22%), verbal (95%) and sexual harassment (16%) [15]. There was also, anothere study conducted in two health region of Gambia reported 62.1% of the participants experienced violence, and 17.2%, 59.8% and 10% of them reported physical, verbal and sexual harassment respectively [16]. Much less (9%) physical violence was observed in Ghana [17]. A study conducted in Nigeria on three selected hospitals, 66% of nurses encountered WPV, and 55% verbal, 8.5% bullying and 6% sexual harassment was reported [18]. Another study was conducted in a teaching university hospital in Nigeria revealed that the magnitude of WPV was a little less than the previous study, and the magnitudes were, 15.3%, 42.9%, 7% and 2.3% of physical, verbal, bullying and sexual harassment respectively [19]

In Ethiopia, directives regarding occupational safety and health (OSH) were to be provided by the Ministry of Labor and Social Affairs (MoLSA). Under the Ethiopian labour proclamation of No. 377/2003, article 92, employers have the legal obligation to protect the health and safety of their workers. But there was no national OSH policy and professionally established body or association, that deals with how the incident should be handled and monitored [20]. Research evidence is very crucial to inform policy for the prevention and control of WPV. Nevertheless, little is known about the prevalence and associated factors of WPV against Nurses in eastern Ethiopia. Therefore, this study aimed to assess the prevalence of WPV and its associated factors among nurse professionals working at public hospitals in eastern Ethiopia.

## Methods

### Study design and setting

Hospital-based cross-sectional study design was conducted at public hospitals of Harari Regional State and Dire Dawa City Administration of eastern Ethiopia from 1^st^ June to 12^th^ July 2021. The study was conducted in six hospitals found in the Eastern part of Ethiopia: three from Harari Regional State (namely; Hiwot Fana Specialized University Hospital (HFSUH), Jugal General Hospital [21] and Harari Federal Police Hospital [22]) and three from Dire Dawa City Administration (namely; Dilchora Referral Hospital (DRH), Sabian General Hospital (SGH) [23] and East Command Level 3 Hospital (ECL3H) [24]). In the 2021 quarterly report from each hospital the total number of nurses were 306 at HFSUH, 173 at JGH [21], 58 at HFPH [22], 162 at DRH, 103 at SGH [23], and 71 at ECL3H [24]. There were 873 nurses in the selected public hospitals of Harari Regional State and Dire Dawa City Administration. The facilities were selected considering the referral destination in the country, and many nurses are deployed in the facilities.

### Study participants and sampling methods

**Fig. 1:**
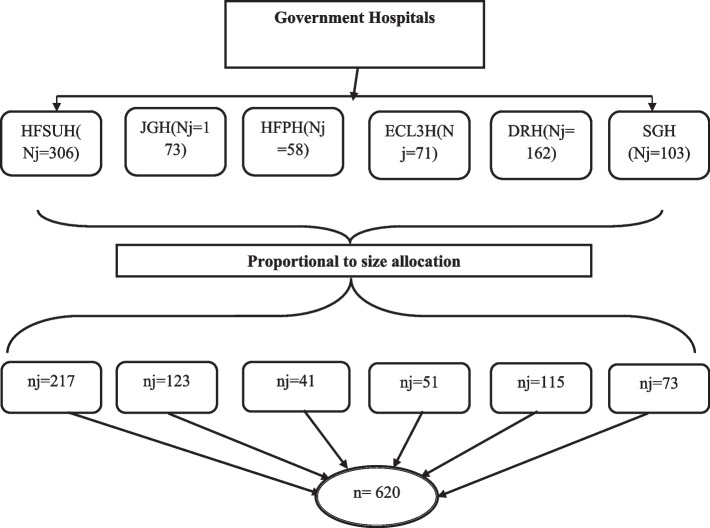
Schematic presentation of the sampling method 2021**.** Key: HFSUH –Hiwot Fana Specialized University Hospital; JGH – Jugol General Hospital; HFPH – Harari Federal Police Hospital; ECL3H - East Command Level 3 Hospital; DRH – Dilchora Referral Hospital; SGH – Sabian General Hospital. Ni- total number of nurses in the Hospital; ni- number of sampled nurses from the hospital; n – Total Sample size

Nurses who were actively working for at least a year in public hospitals of Harari Regional State and Dire Dawa City Administration were included in the sampling procedure. Nurses who were on maternity leave, sick leave, or annual leave during the study period were not included in the study. The sample size was calculated by double population proportion formula using Epi-Info version 7.2.4.0 for individual factors at 99% significance level with 1% margin of error, 80% power and 1:1 ratio of exposed (33%) to unexposed (17.9%) and odds ratio (2.02) [25]. Adding 10% non-respondent rate (*n* = 564), finally the sample size was 620. Then the calculated sample size was proportionally allocated to the number of nurses working in each targeted hospital (Fig. 1). The sampling frame was constructed by listing all nurses who were working in targeted hospitals. Then study participants were selected by using a simple random sampling technique as per the allocated sample size.

### Data collection tool and procedure

Data were collected using a structured and pretested self-administered questionnaire. The questionnaire was adapted from International Labor Office (ILO), International Council of Nurses (ICN), World Health Organization (WHO), and Public Services International [26] country-based survey questionnaire [26]. The questionnaire comprised of four parts; socio-demographic and occupational characteristics which included more than 17 questions; physical WPV which included more than 3 questions; psychological WPV (including; verbal abuse, bullying/mobbing and sexual harassment) which included 12 questions each and health sector employer-related questions which included more than 6 questions. Data collection was facilitated by six nurses who worked in respective government hospitals. These data collectors were recruited due to their previous experiences in data collections. Then those nurses used as a data collector were excluded from the sampling frame. Brief orientation was given to data collectors on the study objectives, sampling, consent, data privacy, and checking for data clarity and completeness by the principal investigator. Collected data were checked for completeness and consistency and the overall quality assurance measures. To assure the quality of the data, pretest was done in Haramaya general hospital among 10% of the total sample size, a two-day training was given to the data collectors and the supervisors. The Investigators supervised the data collection process. To minimize the risk of Covid-19 transmission, data collectors strictly followed all Covid-19 prevention precautions.

### Outcome variable measurements

Workplace violence(WPV): is considered when a nurse had experienced at least one form of physical, psychological and or sexual violence (verbal abuse, sexual harassment, bullying/mobbing) within the last 12 months preceding the survey [27].

Physical violence: When a nurse experienced at least one form of physical violence items, such as beating, kicking, slapping, stabbing, shooting, pushing, biting, spitting on and/or pinching, from others.

Psychological violence (Emotional abuse): Any intentional use of power, including the threat of physical force, against a nurse that might include; verbal abuse or bullying/Mobbing or sexual harassment or concurrent.

### Data processing and analysis

Collected data were entered into EpiData 3.1 and exported to SPSS (version 28) for cleaning and analysis. Descriptive statistics were carried out to compute frequency, proportion, median and the inter-quartile range. Bivariate and multivariable logistic regression analyses were performed to examine the association between the explanatory variables and the outcome variable (WPV). There were 23 explanatory variables, and among those 11 had a *p*-value < 0.25 in bivariate analysis and then they had been included in multivariable logistic regression analysis. Assumptions for logistic regression such as; multicollinearity (correlation coefficient; VIF, tolerance) between the explanatory variables was checked. Hosmer–Lemeshow goodness-to-fit model was tested for multivariate fitness and the result was 0.446. In multivariate, Adjusted Odds Ratio (AOR) with 95% Confidence Interval (CI) at a *P* value of less 0.05 was used to declare statistically significant association (Table 4).

## Results

### Socio-demographic characteristics of the study participants

Out of the total estimated sample (*n* = 620), 603(97.3%) of the study subjects gave consent and completed the self-administered questionnaire. The median ages of the study participants were 30 years (IQR ± 9). More than half (58.0%, *n* = 350) of them were females, and 369 (61.2%) of them were married. Half (50.4%, *n* = 304) of them belonged to Orthodox Christianity by religion. The majority of them (82.1%, *n* = 495) had a Bachelor of Science degree in Nursing (Table 1).

**Table 1:** Socio-demographic characteristics of nurses working in government hospitals, eastern Ethiopia, 2021 (*n* = 603)

Variable	Categories	n	%
Age	≤ 25	77	12.8
	26–35	374	62.0
	≥ 36	152	25.2
Sex	Female	350	58
	Male	253	42
Marital status	Single	210	34.8
	Married	369	61.2
	Co-habiting	1	.2
	Separated/divorced	20	3.3
	Widowed	3	.5
Religion	Muslim	169	28.0
	Orthodox	304	50.4
	Protestant	113	18.7
	Other^a^	17	2.8
Ethnicity	Oromo	243	40.3
	Amhara	221	36.7
	Harari	25	4.1
	Other^b^	114	18.9
Educational status	Diploma	91	15.1
	Bachler	495	82.1
	Masters	17	2.8

^a^ Catholic, Waqefata, Jehovah’s Witness, un-affiliated

^b^ Tigray, Somali, Gurage, Sidama

#### Workplace related characteristics

Eighty-eight percent (*n* = 535) of the nurses had mainly been involved in the clinical service provider role. Almost all (95.9%, *n* = 578) of them worked in shifts and only 93 (15.4%) of them did not work in night shifts. The majority (88.2%, *n* = 532) of nurses had routine and direct physical contact with patients (such as washing, turning and lifting). Only one in five (21.6%) of nurses worked in specialized units. Three-fourths (75.6%, *n* = 456) of nurses reported different extents of being worried about WPV at their health facilities. More than half (59.7%, *n* = 360) of nurses did not aware of the existence of the reporting procedures of WPV attempts or incidents (Table 2).

**Table 2:** Workplace related characteristics among nurses working in government hospitals, eastern Ethiopia, 2021 (*n* = 603)

Variables	Category	n	%
Present position	Nurse head	68	11.3
	Service provider	535	88.7
Work experience	1–4	194	32.2
	5–9	204	33.8
	≥ 10	205	34.0
Shift work	Yes	578	95.9
	No	25	4.1
Night shift (18h00-01h00)	Yes	510	84.6
	No	93	15.4
Routine direct physical contact	Yes	532	88.2
	No	65	10.8
Department working in	Ambulatory/OPD	86	14.3
	General medicine	70	11.6
	General surgery	53	8.8
	Psychiatric	29	4.8
	Emergency	53	8.8
	Pediatrics	57	9.5
	GYN/OBS	93	15.4
	Specialized unit/clinic^a^	133	22.1
	Other^b^	29	4.8
Number of staffs	1–4	522	86.6
	5–9	68	11.3
	≥ 10	13	2.2
How worried about violence	Not worried	147	24.4
	Little worried	165	27.4
	Moderately worried	169	28.0
	Much worried	79	13.1
	Very worried	43	7.1
Reporting procedures	Yes	243	40.3
	No/don't know	360	59.7

*GYN/OBS* Gynecology/obstetrics, *OPD* Outpatient department

^a^ ICU/NICU, Orthopedics, OR, Recovery, Burn, ART/TB/VCT, ANC, MCH, EPI, Nutrition, Dialysis

^b^ Liaison, Oncology

#### Workplace violence against nurses

**Fig. 2:**
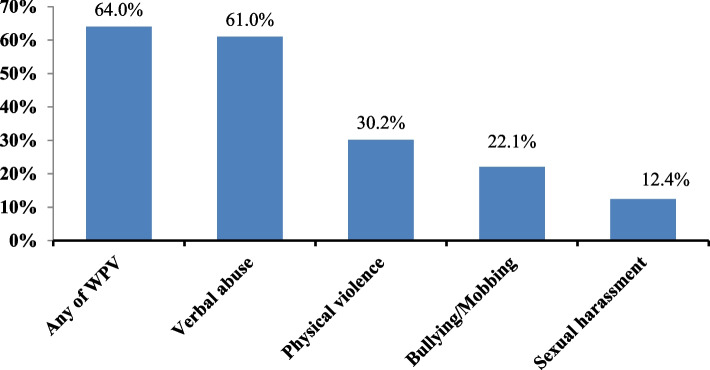
Prevalence of WPV and its types among nurses working in government hospitals, eastern Ethiopia, 2021(*n* = 603) *Multiple responses were possible

The prevalence of WPV against nurses for the last 12 months preceding the survey was 64.0% (95% CI: 60.2–67.7%) (Fig. 2).

### Nurses’ responses towards workplace violence

Out of the total study participants, six-in-tens (61.2%, *n* = 369) of nurses reported that there was a “no or don’t know” institutional policy regarding WPV. Approximately three-fourth (72%, *n* = 434) of nurses were aware of some forms of measures that exist to deal with WPV in their organization. Of these, more than half of nurses reported security measures and changed shifts or rotations were some among the measures. Nevertheless, more than half (51.7%, *n* = 312) were not satisfied with the institution measures **(**Table 3).

**Table 3:** Response to institutional characteristics among nurses working in government hospitals, eastern Ethiopia, 2021 (*n* = 603)

Variable	Category	n	%
Institution policy	Yes	234	38.8
	No/don't know	369	61.2
Specific policy on			
	Health & safety	204	33.8
	Physical violence	151	25.0
	Verbal abuse	141	23.4
	Sexual harassment	120	19.9
	Bullying/mobbing	84	13.9
Measures exist	Yes	434	72.0
	No/ don't know	169	28.0

### Factors associated with workplace violence against nurses

In the bivariate analysis, 23 explanatory variables were considered for analysis, but 11 explanatory variables (age categorized, educational level, work experience, night shift, working department/unit, number of staff, being worried of violence, claimed “absence/not-aware of the reporting procedure”, being a witness of physical violence, claimed “absence/not-aware” of institutional policy and being satisfied with organizational/institutional measures) showed association with WPV. Whereas, in the multivariate analysis at 95% CI and *p*-value < 0.05, only five of the explanatory variables were found to be significantly associated with WPV.

Nurses who were working in surgical (AOR: 2.30, 95%CI: 1.01–5.26), psychiatric wards (AOR: 3.06, 95%CI: 1.11–8.46), medical wards (AOR: 5.20, 95%CI: 2.40–11.27); working in an emergency (AOR: 3.62, 95%CI: 1.46–8.98) were more likely to encounter violence than working in specialized units (Orthopedics, OR, Recovery, Burn, ANC and others). Nurses who were worried about violence (AOR: 1.71, 95%CI: 1.09–2.69) were more likely to encounter violence than those nurses who reported not being worried. Nurses who witnessed physical violence (AOR: 5.31, 95%CI: 3.28–8.59) were 5 times more likely to experience violence than those nurses who reported they did not witness physical violence. Nurses who claimed “absence/not-aware” of reporting procedure (AOR: 2.24, 95%CI: 1.45–3.46) were 2 times more likely to be exposed to WPV than those nurses who were aware of the reporting procedure of violence. Nurses who reported, “absence/not-aware” of institutional policies on WPV (AOR: 2.68, 95%CI: 1.73–4.13) were 2 times more likely to be exposed to WPV than those nurses who reported, “presence/aware” of the presence of institutional policies of violence (Table 4).

**Table 4:** Factors associated with WPV among nurses working in government hospitals, eastern Ethiopia, 2021 (*n* = 603)

Variable	Response category	Workplace violence		COR (95% CI)	*P*-value	AOR (95% CI)	*P*-Value
		Yes	No				
Age	≤ 25	38	39	0.75 (0.43, 1.3)	0.301	1.37 (0.58, 3.24)	0.472
	26–35	262	112	1.80 (1.22, 2.65)	0.003	1.71 (0.92, 3.14)	0.088
	≥ 36	86	66	1		1	
Educational level	Diploma	67	24	1.69 (1.03, 1.78)	0.004	1.62 (0.88, 2.98)	0.118
	Degree &above	319	193	1	0.026	1	
Work Experience	1–4	110	84	0.79 (0.53, 1.18)	0.243	0.66 (0.34, 1.28)	0.219
	5–9	148	56	1.59 (1.05, 2.41)	0.030	1.12(0.60, 2.08)	0.730
	≥ 10	128	77	1		1	
Night shift	Yes	339	171	1.94 (1.24, 3.03)		1.33(0.75, 2.35)	0.317
	No	47	46	1	0.004	1	
Department	Ambulatory/OPD	47	39	0.99 (0.59, 1.67)	0.965	1.39(0.73,2.65)	0.319
	Medical ward	53	17	2.56 (1.37, 4.79)	0.003	5.20(2.40,11.27)*	**0.000**
	Surgical ward	38	15	2.08 (1.06, 4.07)	0.033	2.30(1.01, 5.26)*	**0.049**
	Psychiatric	18	11	1.34 (0.60, 3.02)	0.477	3.06(1.11, 8.46)*	**0.031**
	Emergency	42	11	3.13 (1.51, 6.52)	0.002	3.62(1.46, 8.98)*	**0.005**
	Pediatrics	32	25	1.05 (0.57, 1.93)	0.875	1.70 (0.81, 3.61)	0.163
	Gyn/Obs	67	26	2.11 (1.22, 3.66)	0.007	2.010.99,4.07 ()	0.055
	Specialized unit	89	73	1		1	
Number of staffs	1–4	326	196	0.50 (0.14,1.84)	0.295	0.35(0.09, 1.44)	0.147
	5–9	50	18	0.83 (0.21,3.37)	0.798	0.53(0.11, 2.54)	0.425
	≥ 10	10	3	1		1	
Worried of violence	Worried	311	145	2.06 ( 1.41,3.01)	< 0.001	1.71(1.09,2.69)*	**0.020**
	Not worried	75	72	1		1	
Reporting procedure	Yes	134	109	1	< 0.001	1	** < 0.001**
	No/don't know	252	108	1.90 (1.35, 2.66)		2.24(1.45, 3.46)	
Witnessed physical violence	Yes	190	30	6.04 (3.92, 9.33)	< 0.001	5.31(3.28,8.59)*	** < 0.001**
	No	196	187	1		1	
Institution policy	Yes	117	117	1	< 0.001	1	** < 0.001**
	No/don't know	269	100	2.70 (1.91.,3.80)*		2.68 (1.73,4.13)*	
Satisfied with measures	Yes	172	119	0.66 (0.47,0.93)	0.016	0.77 (0.51,1.18)	0.234
	No	214	98	1		1	

*significantly associated

## Discussion

The study determined the prevalence of WPV and its associated factors among nurse professionals. Nearly two-thirds (64%) of nurses had experienced WPV in the last 12 months. More than a quarter (28.2%) of nurses experienced both physical and psychological forms of violence. Less than a tenth (7.1%) of them experienced all forms of WPV in the last 12 months. The main perpetrators in all types of WPV were Relatives of patients/clients and patients/clients themselves. In this study; the department/working unit, being worried about violence, being witnessing physical violence, reporting procedures and institution policy on WPV were significantly associated.

In line with the prevalence in this study, a similar finding was noted in Nepal(64.5%) [28], Bangladesh (64.2%) [29], Gambia (62.1%) [16] and south-west Nigeria (67%) [18]. On the other hand, a lower prevalence was noted in the studies conducted in the USA (46%) [30], Hong Kong (44.3%) [31], Italy (42%) [32], and in 5 European countries (54%) [33]. This prevalence gap is possibly due to the socio-economic difference noted between the developed and developing countries and it might be related to the gap between the services demanded by the service user and the service provided by the nurses. Additionally, there could be burnouts related to the overload of nurses in providing care to the patients, which indirectly affects the relationship of the nurses with the patients/patient relatives. This are some of the most influential factors affecting the relationship between the service provider and service user in developing countries like that of Ethiopia. The finding of this study is also seemingly higher than studies conducted in Amhara Regional State (26.7%) [25] and Hawasa City Administration (29.9%) [8] of Ethiopia. This huge gap in contrast to this study is due to the operational definitions. Both forms of WPV of this study were similar to the above studies. In the same tone, a lower prevalence was noted in Gamo Gofa zone of southern Ethiopia (43.1%) [27]. This might be due to the socio-cultural differences and/or high prevalence of psychoactive substance use in this study area that might influence individuals to be more impulsive and aggressive. A systematic review and meta-analysis done among nurses in the South-East Asian and Western Pacific region revealed that the pooled prevalence of WPV was 58% [34]. The difference with this pooled prevalence might be due to the convergence of multiple studies which vary significantly in their magnitude. In the other extreme, a higher prevalence was reported in Greek (76%) [35] and Malawi (71%) [15]. This gap is to a part contributed to the high prevalence of verbal abuse in those countries, which is easy to be manifested in one’s aggression and/or might be due to the less weight given in this study area and using smaller sample size in these studies. A higher prevalence was also noted in the Oromia Regional State of Ethiopia (82.2%) [36]. This might be due to the nature of the study setting, the study was done in a referral hospitals in Oromia with a higher number of very frustrated patients and relatives attending services; in that high patient flow aggregated with long waiting time for service tempted the perpetrators to respond violently, mainly through aggressive words.

It is evidenced that, WPV is common among health care work force, particularly among nurses which can lead to serious diverse negative consequences among the nurses or patients/patient families. In sub-Saharan Africa, WPV was reported in many countries, where there are limited health care professionals the consequence on the health care system would be paramount. This high prevalence of violence would finally become a public health threat. There for it is important to use the evidences generated to formulate strategic plans, like raising awareness, frequent incidence reporting and reviewing, adequate staffing, regular training in early identification of violence, which will help to decrease WPV among health care professional [37, 38].

The clinical setting nurses working in were more likely riskier in the departments of surgery, emergency, psychiatry and medical wards than the specialized units. This is in line with studies conducted in Hawasa City Administration [8] and Oromia Regional State [36]. This might be related to the relatively high number of patient admissions, the unstable & violent nature of patients and coupled with a stressful working environment. It is recommended to have adequate staffing, adequate working & visitors spacing, restricting public access and educating de-escalation and risk management skills on predisposed working departments.

Being worried about violence and being witnessing physical violence incidents were significantly associated with WPV. These factors were consistent with the studies conducted in Brazil and Jordan [39, 40]. This might be indicating that those who were worried and witnessing violence were working in a clinically predisposed work environment. Studies revealed that the worry about violence is associated with a disabling mental disorder, anxiety disorder [41]. This indicated that those nurses who worried about violence at work place are more likely to experience mental disorder.

The odds of WPV were higher in nurses who reported ‘absence/not-aware of reporting procedure & institutional policies’ on WPV. These factors were noted in a study conducted in Saudi Arabia [42]. This might be related to the absence of a formal reporting procedure might undermine the prevalence of the incident and was unable to take proper measures to minimize or prevent the incident of violence accordingly [12]. In those institutions where organizational polices are in place, there is a significant reductions of risk of violence and expression prohibited violent behaviors [43]. The impact of WPV is paramount, it affects the organization as well as the workers. The cost of WPV is higher in organizations where WPV is higher. Therefore organizations have responsibility to prevent violence first and foremost by creating and sustaining a positive work culture where people are treated respectfully by the management, co-workers and there should be a mechanism where good work is recognized and conflicts are resolved effectively as soon as they arise. Organizations with cultures which support fair working conditions and zero tolerance for workplace aggressions have been shown to help to mitigate the WPV [44].

Finally, piece of literatures revealed that there were different factors associated with WPV ranging from individual factors to societal factors [2]. Existing evidence categorized the factors associated with WPV into three major categories; such as individual/personal factors, organizational/workplace factors and environmental/situational factors [36, 42, 45]. These factors might be positively or negatively influenced in the covid-19 pandemic. A study conducted in Israel reported that there is a decrease in incidence of workplace violence among hospital staffs [46]. On the contrary, studies conducted in USA noted an increase in incidence of violence during the pandemic [47, 48]. Another study conducted in china revealed that there was an increase in the incidence of WPV during the covid-19 pandemic. Security measures were proved to have significant effect in reducing the WPV [49]. Nevertheless, there is no identified evidence that assessed the impact of Covid-19 on workplace violence among health professionals in sub-Saharan Africa and Ethiopia in particular. Considering the global context, it might have a significant impact on the prevalence of WPV in Ethiopia, particularly in the study area.

### Limitations/weaknesses of the study

The study design was a cross-sectional design, which may not allow making establishing a causal relationship between explanatory variables and the dependent variable. Although the study was a prevalence study, still might be prone to recall bias and social desirability bias due to some personal sensitive questions, “for example sexual harassment items” which may not reflect the exact extent of the problem. The other limitation might be that we have not tested the psychometric property of the questionnaire, thought we have directly used the English version. Finally, this study was conducted in a limited area in the Eastern part of Ethiopia which might affect the generalizability of the study result. However, due to the fact that, there are similarities of violence against health care providers globally, this might not be considered a problem.

## Conclusions

Nearly two-thirds of nurses had experienced at least one form of WPV in the last 12 months, which is an acceptable high. The incident of WPV against nurses was higher in general patient care wards than in specialized units. Nurses who were worried about violence and did not aware the presence of reporting procedure were associated with WPV. Nurses being witnessed WPV so far, and did not aware of the existence of institutional policy against WPV or not were the associated factors. We suggest that stakeholders (physicians, nurses, administrators, managers, scientific societies, security bodies, political decision makers and others) could work on early risk identification and management of violent incidents, establish violence reporting and sanction mechanisms using contextual strategies to prevent WPV against nurse professionals. Specially, the alliance between health professionals and security personnel have a significant role in minimizing aggression against health professionals [50]. Use of simple and rapid user friendly reporting technologies could contribute in the reduction of WPV against nurse professionals [51]. Qualitative studies might be helpfull to solidify the results obtained from quantitative studies, such as this, to find possible interventions and solutions in management of aggression in nursing areas [50–52].

## Supplementary Information

**Additional file 1.**

## Data Availability

The participants de-identified data used for the current study will be available upon submitting a reasonable request from the corresponding author (DT) in either SPSS or Stata format and as per the permission obtained from core project principals (HL, NA and AS).

## Abbreviations

ICN: International Council of Nurses
ILO: International Labor Office
WPV: Public Services International [26], Workplace Violence
WHO: World Health Organization
