# Supplementary material for: Workplace violence and its associated factors among nurses working in public hospitals of eastern Ethiopia: a cross-sectional study
Source: BMC Nurs. 2022 Nov 7;21:300. doi: 10.1186/s12912-022-01078-8 (PMC9638229; doi:10.1186/s12912-022-01078-8)
Supplement: Supplementary file 1 — Additional file 1. [file 12912_2022_1078_MOESM1_ESM.docx]

## Questionnaire used to assess workplace violence and associated factors among nurses working in public hospitals of eastern Ethiopia

## Please complete the questionnaire by either Circling boxes or writing in the spaces provided.

**[PART A]. PERSONAL AND WORKPLACE DATA**

| **PD 1** | Where do you work? | 1. Dire-Dawa 2. Harar | |
| --- | --- | --- | --- |
| **PD 2** | Which institution do you work in? | 1. Dilchora General Hospital  2. Sabian General Hospital  3. Hiwot Fana specialized university hospital  4. Jugal General Hospital  5. East Command Level 3 Hospital  6. Harari Federal Police Hospital | |
| **PD 3** | What is your age? | ________ (years) | |
| **PD 4** | Sex: | 1. Female 2. Male | |
| **PD 5** | What is your marital status? | 1. Single 2. Married 3. Co-habiting 4. Separated /Divorced 5. Widow/widower | |
| **PD 6** | What is your Religion? | 1. Muslim 2. Orthodox 3. Protestant  4. Other (Please, specify)___________ | |
| **PD 7** | What is your Ethnicity? | 1. Oromo 2. Amhara 3. Harari  4. Other (Please, specify)___________ | |
| **PD 8** | What is your Educational status? | 1. Diploma Nurse 2. BSc Nurse 3. MSc Nurse | |
| **PD 9** | Which category best describes your present position? | 1. Ward-head/nurses head 2. Staff/service provider | |
| **PD 10** | How many years of work experience in the health sector do you presently have? | ______ | |
| **PD 11** | Do you work in shifts? | 1. Yes 2. No | |
| **PD 12** | Do you work anytime between 18h00 (6 PM) and 01h00  (7 AM)? | | 1. Yes 2. No |
| **PD 13** | Do you interact with patients/clients during your work? | | 1. Yes 2. No   (If No, please go to question **PD 14**) |
|  | 13.1 Do you have routine direct physical contact (washing, turning, and lifting) with Patients/clients? | | 1. Yes 2. No |
|  | 13.2 The sex of the patients you most frequently work with are: | | 1. Female 2. Male 3. Both Female & Male |
|  | 13.3 Please indicate if you spend more than 50% of your time working with any of the following  type of specialties: | | 1. Physically disabled 5. Psychiatric  2. Mentally ill 6. Mother/child care  3. Terminally ill 7. Geriatric  4. HIV/AIDS  8. Other (please specify)____________ |

| **PD 14** | Where do you spend most of your time (more than 50%) in your main job?  (Please choose the work setting that describes it best) | 1. Ambulatory/OPD 5. Emergency  2. General medicine 6. Pediatrics  3. General surgery 7. Gyn/Obs  4. Psychiatric  8. Specialized unit (ICU, Burn, Orthopedics)  9. other (please, specify)_____________ |
| --- | --- | --- |
| **PD 15** | The number of staff present in the same work setting with you during most (more than 50%) of your work time is? | _________ |
| **PD 16** | How worried are you about violence in your current workplace? (Please rate:1 = not worried, 2= little worried, 3=moderately worried, 4=much worried & 5 = very worried) | 1 2 3 4 5 |
| **PD 17** | Are there procedures for the reporting of violence in your workplace? | 1. Yes 2. No   (If No; please go to [**PART B]**) |
|  | 17.1 If YES, do you know how to use them? | 1. Yes 2. No |
|  | 17.2 Is there encouragement to report workplace violence? | 1. Yes 2. No (If NO, please go to [**PART B]**) |
|  | 17.3 If YES, by whom: | 1. Management / employer 2. Colleagues  3. Union 4. Association  5. Own family / friends 6. Other, please specify:___________ |

**[PART B]. PHYSICAL WORKPLACE VIOLENCE**

**Glossary**

**Physical violence**; refers to the use of physical force against another person or group that results in physical harm, sexual or psychological harm. It can include beating, kicking, slapping, stabbing, shooting, pushing, biting, and/or pinching, among others

**Assault/Attack**; Intentional behavior that harms another person physically, including sexual assault (i.e. Rape)

| **PV 1** | In the **last 12 months**, have you been physically attacked in your workplace? | 1. Yes 2. No   (If No, please go to question **PV 2**) |
| --- | --- | --- |
|  | - 1. If yes, please think of the last time that you were physically attacked in your place of work.   How would you describe this incident? | 1. Physical violence without a weapon/Object  2. Physical violence with a weapon/Object |
|  | 1.2 Do you consider this to be a typical/common incident of violence in your workplace? | 1. Yes 2. No |
|  | 1.3 Who attacked you? | 1. Patient/client 2. Relatives of patient/client  3. Staff member/co-worker 4. Management /Supervisor  5. Physician 6. General public  7. Other( please; specify)_________ |
|  | 1.4 Where did the incident take place? | 1. Inside health institution or facility  2. Outside (on way to work / health visit / home) |
|  | 1.5 At which time did it happen? | 1). 07.00h. - before 13.00 h. 2). 13.00 h. – before 18.00 h.  3). 18.00h. – before 24.00 4). 24.00h - before 07.00h  5). Don’t remember |
|  | 1.6 Which day of the week did it happen? | 1. Weekdays 2. Weekend 3. Don’t remember |

|  | - 1. How did you respond to the incident? (Please tick all relevant answers) | 1. took no action 2. tried to pretend it never happened  3. told the person to stop 4. tried to defend myself physically  5. told friends/family 6. sought counseling  7. told a colleague 8. reported it to a senior staff member  9. transferred to another position | |
| --- | --- | --- | --- |
|  | 1.8 Do you think the incident could have been prevented? | 1. Yes 2. No | |
|  | 1.9 Were you injured as a result of the violent incident? | 1. Yes 2. No (If NO, please go to question **1.11**.) | |
|  | 1.10 IF YES, did you require formal treatment for the injuries? | 1. Yes 2. No | |
|  | 1.11 Listed below are a list of problems and complaints that people sometimes have in response  to stressful life experiences like the event that you suffered. For each item, please indicate how bothered you have been by these experiences since you were attacked. Please tick one option per question. | | |
|  | Since you were attacked, how BOTHERED have you been by: (Please rate: **1 = not at all, 2=a little bit, 3=moderately, 4=Quite a bit & 5 = Extremely**) | | |
|  | (a) Repeated, disturbing memories, thoughts, or images of the attack? | | 1 2 3 4 5 |
|  | (b) Avoiding thinking about or talking about the attack or avoiding having feelings related to it? | | 1 2 3 4 5 |
|  | (c) Being "super-alert" or watchful and on guard? | | 1 2 3 4 5 |

|  | (d) Feeling like everything you did was an effort (to be difficult or painful to do)? | 1 2 3 4 5 |
| --- | --- | --- |
|  | 1.12 Did you have to take time off from work after being attacked? | 1. Yes 2. No ( If NO, please go to question **1.13**) |
|  | 1.12.1 If YES, for how long? | 1. One day 2.2-3 days  3. One week 4.2-3 weeks  5.1 month 6.2-6 months  7.7-12 months |
|  | 1.13 Was any action taken to investigate the causes of the incident? | 1. Yes 2. No |
|  | 1.14 What were the consequences for the attacker? | 1. None 2. Verbal warning issued  3. Care discontinued 4. Reported to police  5. Aggressor prosecuted 6. Don't know |
|  | 1.15 Did your employer or supervisor offer to provide you with |  |
|  | Counseling? | 1. Yes 2. No |
|  | Opportunity to speak about/report it? | 1. Yes 2. No |
|  | Other support? | 1. Yes 2. No |
|  | 1.16 How satisfied are you with the manner in which the incident was handled? (Please rate: **1 = very dissatisfied, 5 = very satisfied**) | 1 2 3 4 5 |
|  | 1.17 If you did not report or tell about the incident to others, why not?  (Please tick every relevant answers) | 1. It was not important  2. Felt ashamed  3. Felt guilty  4. Afraid of negative consequences  5. Useless/No action will be taken  6. Did not know who to report to  7. Other (please, specify)___________ |
| **PV 2** | In **the last 12 months**, have you **witnessed** incidents of physical violence in your workplace? | 1. Yes 2. No (if NO, please go to question **PV 3**) |
|  | 2.1 If YES, how often has this occurred in the last 12 months? | 1. Once 2.2-4 times  3.5-10 times 4. Several times a month  5. About once a week 6. Daily |
| **PV 3** | Have you reported an incident of workplace violence in the last 12 months? (witnessed or experienced) | 1. Yes 2. No   (If NO, please go to [**PART C]**) |
|  | 3.1 IF YES, have you been disciplined for reporting an incident of workplace violence? | 1. Yes 2. No |

**[PART C]. PSYCHOLOGICAL WORKPLACE VIOLENCE (Emotional Abuse)**

**GLOSSARY**

**Psychological violence;** is defined as Intentional use of power, including threat of physical force, against another person or group, that can result in harm to physical, mental, spiritual, moral or social development

**Verbal Abuse;** Behavior that humiliates degrades or otherwise indicates a lack of respect for dignity and worth of an individual.

**Sexual harassment;** Any unwanted, unreciprocated and unwelcome behavior of a sexual nature that is offensive to the person involved, and causes that person to be threatened, humiliated or embarrassed. It involved attempts to establish or force sexual relations, to threaten someone into having sex (sexual blackmail), and to offering money, gifts, or privileges in exchange for sexual favors.

| **I. VERBAL ABUSE** | | |
| --- | --- | --- |
| **VA 1** | In **the last 12 months**, have you been verbally abused in your workplace? | 1. Yes 2. No   (If **No**; please go to **BULLYING / MOBBING**) |
| **VA 2** | How often have you been verbally abused in the last 12 months? | 1. All the time 2. Sometimes 3. Once |
| **VA 3** | Please think of the last time you were verbally abused in your place of work. Who verbally abused you? | 1. Patient/client 2. Relatives of patient/client  3. Staff member 4. Management / Supervisor  5. Physician 6. General public  7. Other:___________ |
| **VA 4** | Do you consider this to be a typical/common incident of verbal abuse in your workplace? | 1. Yes 2. No |
| **VA 5** | Where did the verbal abuse take place? | 1. Inside health institution or facility  2. Outside (on way to work/health visit/home |

| **VA 6** | How did you respond to the verbal abuse? (Please tick all relevant answers) | 1. took no action  2. tried to pretend it never happened  3. told the person to stop  4. tried to defend myself physically  5. told friends/family  6. sought counseling  7. told a colleague  8. reported it to a senior staff member  9. transferred to another position | |
| --- | --- | --- | --- |
| **VA 7** | Listed below are a list of problems and complaints that people sometimes have in response to stressful life experiences like the event that you suffered. For each item, please indicate how bothered you have been by these experiences since you were attacked. Please tick one option per question. | | |
|  | Since you were attacked, how BOTHERED have you been? (Please rate: **1 = not at all, 2=a little bit, 3=moderately, 4=Quite a bit & 5 = Extremely**) | | |
|  | (a) Repeated, disturbing memories, thoughts, or images of the attack? | | 1 2 3 4 5 |
|  | (b) Avoiding thinking about or talking about the attack or avoiding having feelings related to it? | | 1 2 3 4 5 |
|  | (c) Being "super-alert" or watchful and on guard? | | 1 2 3 4 5 |
|  | (d) Feeling like everything you did was an effort (to be difficult or painful to do)? | | 1 2 3 4 5 |
| **VA 8** | Do you think the incident could have been prevented? | | 1. Yes 2. No |

| **VA 9** | Was any action taken to investigate the causes of the verbal abuse? (If NO or DON’T KNOW, please go to question **VA 10)** | 1. Yes 2. No 3. don’t know |
| --- | --- | --- |

|  | 9.1 If YES, by whom: (please tick every relevant answers) | 1. Management / Employer  2. Union 3. Association 4. Community group 5. Police  6. Other: please specify_________________ |
| --- | --- | --- |
|  | 9.2 If YES, what were the consequences for the abuser? | 1. None 2. Verbal warning issued  3. Care discontinued 4. Reported to police  5. Aggressor prosecuted  6. Other (please, specify)___________ |
| **VA 10** | Did your employer or supervisor offer to provide you with? |  |
|  | Counseling | 1. Yes 2. No |
|  | Opportunity to speak about/report it | 1. Yes 2. No |
|  | Other support? | 1. Yes 2. No |
| **VA 11** | How satisfied are you with the manner in which the incident was handled? (Please rate: **1 = very dissatisfied, 5 = very satisfied**) | 1 2 3 4 5 |
| **VA 12** | If you did not report or tell about the incident to others, why not? (Please tick every relevant answers) | 1. It was not important 2. Felt ashamed  3. Felt guilty  4. Afraid of negative consequences  5. Useless/No action will be taken  6. Did not know who to report to  7. Other (please, specify)___________ |

**Glossary**

**Bullying / Mobbing**; Repeated and over time offensive behavior through vindictive, cruel, or malicious attempts to humiliate or undermine an individual or groups of employees

| **II. Bullying / Mobbing** | | | | | |
| --- | --- | --- | --- | --- | --- |
| **BM 1** | In the **last 12 months**, have you been bullied / mobbed in your workplace? | | | 1. Yes 2. No   (If No, please go to **SEXUAL HARASSMENT)** | |
| **BM 2** | How often have you been bullied/mobbed in the last 12 months? | | | 1. All the time 2. Sometimes 3. Once | |
| **BM 3** | Please think of the last time you were bullied/mobbed in your place of work. Who bullied/mobbed you? | | | 1. Patient/client  2. Relatives of patient/client  3. Staff member 4. Management / Supervisor  5. Physician 6. General public  7. Other:___________ | |
| **BM 4** | Do you consider this to be a typical/common incident in your workplace? | | | 1. Yes 2. No | |
| **BM 5** | Where did the bully/mobbing take place? | | | 1. Inside health institution or facility  2. Outside (on way to work/health visit/home | |
| **BM 6** | How did you respond to the bully/mobbing?  (Please tick all relevant answers) | | | 1. took no action  2. tried to pretend it never happened  3. told the person to stop  4. tried to defend myself physically  5. told friends/family  6. sought counseling  7. told a colleague  8. reported it to a senior staff member  9. transferred to another position | |
| **BM 7** | Listed below are a list of problems and complaints that people sometimes have in response to stressful life experiences like the event that you suffered. For each item, please indicate how bothered you have been by these experiences since you were attacked. (Please tick one option per question) | | | | |
|  | Since you were attacked, how BOTHERED have you been by: (Please rate: **1 = not at all; 5 = Extremely**) | | | | |
|  | (a) Repeated, disturbing memories, thoughts, or images of the attack? | | | | 1 2 3 4 5 |
|  | (b) Avoiding thinking about or talking about the attack or avoiding having feelings related to it? | | | | 1 2 3 4 5 |
|  | (c) Being "super-alert" or watchful and on guard? | | | | 1 2 3 4 5 |
|  | (d) Feeling like everything you did was an effort? | | | | 1 2 3 4 5 |
| **BM 8** | Do you think the incident could have been prevented? | | | | 1. Yes 2. No |
| **BM 9** | Was any action taken to investigate the causes of the bullying/mobbing?(If No or Don’t know, please go to question **BH 10**) | | | | 1. Yes 2. No 3. Don’t know |
|  | 9.1 If YES, by whom | | | | 1. Management / Employer  2. Union 3. Association  4. Community group 5. Police  6. Other: please specify________ |
|  | 9.2 If YES, what were the consequences for the person who bullied/mobbed you? | | 1. None 2. Verbal warning issued  3. Care discontinued 4. Reported to police  5. Aggressor prosecuted  6. Other: please specify_________ | | |
| **BM 10** | Did your employer or supervisor offer to provide you with? | | | |  |
|  | Counseling | | | | 1. Yes 2. No |
|  | Opportunity to speak about/report it | | | | 1. Yes 2. No |
|  | Other support? | | | | 1. Yes 2. No |
| **BM 11** | How satisfied are you with the manner in which the incident was handled?  (Please rate: **1 = very dissatisfied, 5 = very satisfied** | | | | 1 2 3 4 5 |
| **BM 12** | If you did not report or tell about the incident to others, why not? (Please tick every relevant answers) | 1. It was not important 2. Felt ashamed  3. Felt guilty 4. Afraid of negative consequences  5. Useless/No action be taken 6. Did not know who to report to  7. Other, please specify:___________ | | | |

| **III. SEXUAL HARASSMENT** | | |
| --- | --- | --- |
| **SH 1** | In **the last 12 months**, have you been sexually harassed in your workplace? | 1. Yes 2. No   (If No, please go to **Section C)** |
| **SH 2** | How often have you been sexually harassed in the last 12 months? | 1. All the time 2. Sometimes 3. Once |
| **SH 3** | Please think of the last time you were sexually harassed in your place of work. Who sexually harassed you? | 1. Patient/client  2. Relatives of patient/client 3. Staff member  4. Management / Supervisor 5. Physician  6. General public 7. Other:___________ |
| **SH 4** | Do you consider this to be a typical/common incident of sexual harassment in your workplace? | 1. Yes 2. No |
| **SH 5** | Where did the sexual harassment take place? | 1. Inside health institution or facility  2. Outside (on way to work/health visit/home |
| **SH 6** | How did you respond to the sexual harassment?  (Please tick all relevant answers) | 1. took no action  2. tried to pretend it never happened  3. told the person to stop  4. tried to defend myself physically  5. told friends/family  6. sought counseling  7. told a colleague  8. reported it to a senior staff member  9. transferred to another position |

| **SH 7** | Listed below are a list of problems and complaints that people sometimes have in response to stressful life experiences like the event that you suffered. For each item, please indicate how bothered you have been by these experiences since you were attacked. Please tick one option per question | | |
| --- | --- | --- | --- |
|  | Since you were attacked, how BOTHERED have you been by:(Please rate: **1 = not at all; 5 = Extremely**) | | |
|  | (a) Repeated, disturbing memories, thoughts, or images of the attack? | | 1 2 3 4 5 |
|  | (b) Avoiding thinking about or talking about the attack or avoiding having feelings related to it? | | 1 2 3 4 5 |
|  | (c) Being "super-alert" or watchful and on guard? | | 1 2 3 4 5 |
|  | (d) Feeling like everything you did was an effort (to be difficult or painful to do)? | | 1 2 3 4 5 |
| **SH 8** | Do you think the incident could have been prevented? | | 1. Yes 2. No |
| **SH 9** | Was any action taken to investigate the causes of the sexual harassment? (If No or Don’t know, please go to question **SH 10**) | | 1. Yes 2. No 3. Don’t know |
|  | 9.1 If YES, by whom | 1. Management / Employer 2. Union  3. Association 4. Community group  5. Police 6. Other: please specify________ | |
|  | 9.2 If YES, what were the consequences for the person who harassed you? | 1. None 2. Verbal warning issued  3. Care discontinued 4. Reported to police  5. Aggressor prosecuted 6. Other: please specify_________ | |

| **SH 10** | Did your employer or supervisor offer to provide you with? | | | | | |
| --- | --- | --- | --- | --- | --- | --- |
|  | Counseling | 1. Yes 2. No | | | | |
|  | Opportunity to speak about/report it | 1. Yes 2. No | | | | |
|  | Other support? | 1. Yes 2. No | | | | |
| **SH 11** | How satisfied are you with the manner in which the incident was handled?  (Please rate: **1= very dissatisfied, 5 = very satisfied)** | 1 2 3 4 5 | | | | |
| **SH 12** | If you did not report or tell about the incident to others, why not? (Please tick every relevant box) | 1. It was not important 2. Felt ashamed  3. Felt guilty 4. Afraid of negative consequences  5. Useless/No action will be taken 6. Did not know who to report to  7. Other (please, specify)___________ | | | | |
| **C. HEALTH SECTOR EMPLOYER** | | | | | | |
| **HE 1** | Is there an institution policy on Workplace violence? | | 1. Yes 2. No  (If No, please go to **HE 3**) | | | |
| **HE 2** | Has your employer developed specific policies on: | | | | | |
|  | Health and safety | | | 1. Yes 2. No 3. I don’t Know | | |
|  | Physical workplace violence | | | 1. Yes 2. No 3. I don’t Know | | |
|  | Verbal abuse | | | 1. Yes 2. No 3. I don’t Know | | |
|  | Sexual harassment | | | 1. Yes 2. No 3. I don’t Know | | |
|  | Bullying/Mobbing | | | 1. Yes 2. No 3. I don’t Know | | |
|  | Threat | | | 1. Yes 2. No 3. I don’t Know | | |
| **HE 3** | Is there Measures to deal with workplace violence in your organization? | | | 1. Yes 2. No | | |
| **HE 4** | What measures to deal with workplace violence exist in your workplace? | | | | | |
|  | Security measures (e.g. guards, alarms, portable telephones) | | | | | 1. Yes 2. No |
|  | Improve surroundings (e.g. lighting, noise, heat, access to food, cleanliness, privacy) | | | | | 1. Yes 2. No |
|  | Restrict public access | | | | | 1. Yes 2. No |
|  | Patient screening (to record and be aware of previous aggressive behavior) | | | | | 1. Yes 2. No |
|  | Restrict exchange of money at the workplace (e.g. patient fees) | | | | | 1. Yes 2. No |
|  | Increased staff numbers | | | | | 1. Yes 2. No |
|  | Changed shifts or rotations (i.e. working times) | | | | | 1. Yes 2. No |
|  | Reduced periods of working alone | | | | | 1. Yes 2. No |
|  | Training  (e.g. workplace violence, coping strategies, communication skills, conflict resolution, self-defense) | | | | | 1. Yes 2. No |
| **HE 5** | Are you satisfied with the organizations measures? | | | | 1. Yes 2. No | |
| **HE 6** | What do you think is the contributing factor for you encountering violence? | 1. Long waiting time for service 2. Misunderstanding 3. Stressful emergency situation 4. Patients condition (Under influence of substance e.g chat, alcohol) or mental illness) 5. Lack of prescribed drug 6. Lack of security measures 7. Other (please, specify) ___________________ | | | | |

**THANK YOU!**
